# Unmet needs and new challenges in young women’s sexual and reproductive health and rights: a qualitative study in Chile’s Metropolitan Region

**DOI:** 10.1080/26410397.2025.2558272

**Published:** 2025-09-15

**Authors:** Alexandra Obach, Michelle Sadler, Consuelo Robledo, Ciara Wright, Báltica Cabieses

**Affiliations:** a Associate Professor, Centro de Salud Global Intercultural, Universidad del Desarrollo, Santiago, Chile; b Adjunct Researcher, Departamento de Historia y Ciencias Sociales, Facultad de Artes Liberales, Universidad Adolfo Ibáñez, Santiago, Chile. *Correspondence:* misadler@edu.uai.cl; c Independent Researcher, Centro de Salud Global Intercultural, Universidad del Desarrollo, Santiago, Chile; d Research Assistant, Birkbeck College, School of Social Sciences, University of London, London, UK; e Professor, Centro de Salud Global Intercultural, Universidad del Desarrollo, Santiago, Chile; Visiting Senior Scholar, Department of Health Sciences, University of York, York, UK

**Keywords:** sexual and reproductive health (SRH), sexual and reproductive rights, young women, health services accessibility, qualitative research, Chile

## Abstract

In Chile, despite important advances in access to contraception and a steady reduction in unplanned pregnancy, longstanding barriers for young people to access sexual and reproductive health (SRH) and rights – rooted in a conservative and religious background – have been highlighted by recent socio-political movements, including the feminist student wave of 2018 and the social uprising of 2019. The COVID-19 pandemic further strained access, leading to the suspension of many in-person services. In this context, we conducted a qualitative study between 2020 and 2023 to explore young women’s perceptions of sexuality and SRH, their interactions with the formal healthcare system, and the diverse resources they engage with to access support and care. 23 in-depth interviews were carried out with heterosexual and non-heterosexual women aged 18–25 in Chile’s Metropolitan Region. The findings reveal a mismatch between young women’s holistic understanding of sexuality – which includes emotional, biological, and political dimensions – and the healthcare system’s reductionist, heteronormative, and risk-based approach, which became more visible after these societal upheavals. As a result, young women may use biomedical SRH services strategically for prescriptions and testing, while seeking more comprehensive support outside the formal system through virtual platforms, health professionals giving online support, and civil society organisations. The study concludes that systemic changes in the health system are needed to bridge these divides and uphold the sexual and reproductive rights of young women in Chile, especially those who identify as non-heterosexual. *DOI: 10.1080/26410397.2025.2558272*

## Plain language summary

Contraception coverage has improved and there is a steady decline in adolescent pregnancies in Chile. But young people still face major barriers to achieving sexual and reproductive health services and rights, in a country where conservative and religious traditions remain influential. Socio-political movements such as the feminist protests in 2018 and the 2019 social uprising exposed how the health system struggles to meet the SRH needs of women, especially non-heterosexual women. These issues worsened during the COVID-19 pandemic, which disrupted many in-person health services. We conducted a qualitative study in 2021–23 with lower-middle class young women aged 18–25 in the Metropolitan Region of Chile. We wanted to find out how they view sexuality and SRH, how they interact with the formal healthcare system, and where they find support. We interviewed 23 women, both heterosexual and non-heterosexual. Our findings show a gap between what young women need and what the healthcare system provides. Participants see their bodies and sexuality in a holistic way, including emotional and political aspects. But the healthcare system mostly focuses on risks and medical issues. Some young women use the healthcare system for services such as contraception, prescriptions or tests. For broader support, they also use online platforms, professionals who provide care online, and civil society organisations. We conclude that Chile’s healthcare services need to treat sexuality in a more holistic and less heteronormative way. A stronger rights-based approach would meet the SRH needs of diverse young women and protect their rights.

## Résumé

Au Chili, malgré d’importantes avancées dans l’accès à la contraception et une baisse constante des grossesses non planifiées, les obstacles de longue date à l’accès des jeunes à la santé et aux droits sexuels et reproductifs, enracinés dans un environnement conservateur et religieux, ont été mis en évidence par les récents mouvements sociopolitiques, notamment la vague féministe des étudiantes en 2018 et le soulèvement social de 2019. La pandémie de COVID-19 a encore restreint l’accès, entraînant la suspension de nombreux services en personne. Dans ce contexte, nous avons réalisé un projet qualitatif entre 2020 et 2023 pour étudier les perceptions de la sexualité et de la santé sexuelle et reproductive (SSR) chez les jeunes femmes, leurs interactions avec le système de santé formel et les diverses ressources auxquelles elles font appel pour accéder au soutien et aux soins. 23 entretiens approfondis ont été menés avec des femmes hétérosexuelles et non hétérosexuelles âgées de 18 à 25 ans dans la région métropolitaine de Santiago du Chili. Les résultats révèlent un décalage entre la compréhension holistique de la sexualité chez les jeunes femmes, comprenant des dimensions émotionnelles, biologiques et politiques, et l’approche réductionniste, hétéronormative et fondée sur les risques du système de santé, rendu plus visible par les récents bouleversements sociétaux. Par conséquent, les jeunes femmes peuvent recourir aux services biomédicaux de SSR de manière stratégique pour des ordonnances et des tests, tout en recherchant un soutien plus complet en dehors du système formel, par le biais de plateformes virtuelles, de professionnels de la santé proposant un appui en ligne et d’organisations de la société civile. L’étude en conclut que des changements systémiques sont nécessaires au sein du système de santé pour combler ces écarts et défendre les droits sexuels et reproductifs des jeunes femmes au Chili, en particulier celles qui s’identifient comme non hétérosexuelles.

## Resumen

En Chile, a pesar de importantes avances en el acceso a métodos anticonceptivos y una reducción sostenida de los embarazos no planificados, las barreras de larga data que enfrentan las personas jóvenes para acceder a la salud sexual y reproductiva (SSR) y derechos en este ámbito, arraigadas en un contexto conservador y religioso, han sido puestas de relieve por recientes movimientos sociopolíticos, tales como la ola estudiantil feminista de 2018 y la revuelta social de 2019. La pandemia de COVID-19 dificultó aun más el acceso y propició la suspensión de muchos servicios en persona. En este contexto, realizamos un proyecto cualitativo entre 2020 y 2023 para explorar las percepciones de mujeres jóvenes en torno a la sexualidad y SSR, sus interacciones con el sistema de salud formal y los diversos recursos que utilizan para acceder a apoyo y servicios de salud. Se realizaron 23 entrevistas en profundidad con mujeres heterosexuales y no heterosexuales entre 18 y 25 años, en la Región Metropolitana de Chile. Los hallazgos revelan una incongruencia entre la comprensión holística de la sexualidad por parte de las jóvenes – que abarca las dimensiones emocionales, biológicas y políticas – y el enfoque reduccionista, heteronormativo y basado en riesgos del sistema de salud, que se volvió más visible después de los recientes movimientos sociopolíticos. Por consiguiente, las mujeres jóvenes a veces utilizan servicios biomédicos de SSR estratégicamente para obtener recetas médicas y pruebas, a la vez que buscan apoyo más integral fuera del sistema formal por medio de plataformas virtuales, profesionales de salud que brindan apoyo en línea y organizaciones de la sociedad civil. El estudio concluye que se necesitan cambios sistémicos en el sistema de salud para superar estas brechas y proteger los derechos sexuales y reproductivos de las jóvenes en Chile, especialmente de aquellas que se identifican como no heterosexuales.

## Introduction

Young people’s health is a critical marker of future population health and a key driver of social and economic development worldwide.^1^ Addressing adolescence and youth is particularly complex, as this phase represents a period of heightened vulnerability.^2,3^ Among the various dimensions of health, sexual and reproductive health (SRH) is particularly significant during this period, since individuals form their sexual and emotional identities, navigate sexual relationships, and face heightened risks of morbidity and mortality related to SRH.^4^ Globally, adolescents and young people face significant disparities in accessing healthcare and profound violations of their sexual and reproductive rights, including sexual abuse, child marriage, and limited access to comprehensive sexual education.^5,6^ The consequences of denying young people’s sexuality and rights, particularly for women and girls, are often exacerbated by conservative and traditional forces.^7^ However, despite these pressing needs, healthcare systems often remain ill-equipped to address the unique requirements of adolescents and young people, as persistent prejudices highlight the urgent need for reform to safeguard their rights.^7,8^

This is especially true in regions like Latin America, where conservative views on sexuality – often heavily influenced by the power of Catholic and Pentecostal Churches – are deeply embedded.^9^ Frequently neglecting their sexual rights and hesitating to recognise young people’s sexual autonomy, SRH services are mainly focused on the prevention of unplanned pregnancies and sexually transmitted infections (STIs).^10^ This narrow approach has created a significant disconnect between the SRH services offered and the real needs of young people.^11,12^ Efforts to reduce HIV/AIDS among youth have advanced more slowly than in other regions,^13^ and adolescent pregnancy rates (ages 15–19) remain among the highest in the world.^14^ At the same time, many countries in the region still lack comprehensive sexual education programs and sufficient access to contraception and safe abortion services.^15^

In Chile, there have been positive outcomes in SRH from targeted health programs. Since 2008, Chile's Ministry of Health has introduced strategies to improve adolescent health, including the 2009 launch of Youth-Friendly Health Services in primary care. These services aim to provide accessible, appropriate, and effective care for adolescents aged 10–19, with a focus on SRH and preventing teen pregnancy.^16^ These efforts have led to a steep decline in teenage pregnancy, making Chile’s rate the lowest in Latin America in 2018. Nonetheless, income-based inequalities remain sharp, with a 63-fold higher adolescent pregnancy rate in the poorest compared to the wealthiest income group.^17^ Additionally, STIs, including HIV, have surged, with Chile seeing a 35% increase in new HIV cases over the last decade – the highest rise in the region.^18,19^ Among youth aged 15–19, HIV cases increased by 20% between 2015 and 2017, reaching 9.1 per 100,000, with rates as high as 41.7 per 100,000 for those aged 20–24.^20^ This is exacerbated by Chile’s segmented health system, divided between public and private sectors, which creates barriers to accessing healthcare, including SRH services. Vulnerable youth primarily depend on the public system, while wealthier individuals access private insurance-based care, further reinforcing health inequalities.^21^

Along with the above, comprehensive sexual education remains restricted in the country due to conservative influences, particularly from the Catholic Church, which has long shaped public policy.^22^ Furthermore, in the health sector, youth sexuality is still approached largely from a risk-oriented perspective, which often stigmatises young people and neglects the diversity of their gender and sexual identities and behaviours, thereby undermining the effectiveness of health interventions.^23,24^

This is especially concerning given data showing a substantial increase in young people identifying as LGBTQI+ during the last decade, rising from 3.4% in 2012 to 15.0% in 2022,^25^ a shift influenced by recent political movements reshaping gender dynamics. In 2018, inspired by the #NiUnaMenos and #MeToo movements, Chile lived a feminist “revolution” sparked by reports of harassment and abuse in universities and widespread protests against institutional inaction. This feminist wave, with its central demand “Against the Precarity of Life”, highlighted economic insecurity under capitalism and patriarchy and set the stage for the 2019 social uprising, where the slogan of “dignity” captured widespread frustration with extreme neoliberal policies and deepening inequalities.^26^ Within this context, the structural limitations of the healthcare system in addressing and ensuring SRH care and rights for all women, including non-heterosexual women, became increasingly apparent. These shortcomings were intensified further with the onset of the COVID-19 pandemic, which led to the suspension of many in-person SRH services.^27^

Indeed, an expanding body of research worldwide highlights significant SRH disparities experienced by sexual minority women – such as lesbians and bisexuals – compared to their heterosexual counterparts.^28^ These disparities include lower rates of contraceptive use and higher rates of STI diagnoses, lower rates of screenings, such as Pap (Papanicolaou) smears and mammograms, reduced access to health services, and greater difficulty in finding understanding healthcare providers, compared to heterosexual women.^29,30^

Given the current context, addressing emerging and shifting needs and challenges in young women’s SRH and rights is of critical relevance. This article aims to explore young women’s perceptions of sexuality and sexual health, their interactions with the formal healthcare system, as well as the diverse resources they use for support and care. Gaining this understanding is essential for tailoring services more effectively to meet their specific needs. To achieve these objectives, we conducted a qualitative research study (Fondecyt #11190701) between 2020 and 2023 in three regions of Chile. This article presents findings from a sub-sample of the broader study. Results from other sub-samples have been published elsewhere.^31–33^

## Methods

This methods section follows the consolidated criteria for reporting qualitative studies (COREQ) 32-item checklist.^34^

### Study design and setting

Project Fondecyt #11190701 (2020–2023) aimed to explore how young people, aged 18–25, understand sexuality and sexual health, as well as how they engage with the healthcare sector and other resources in shaping their support and therapeutic choices. The project followed a qualitative design, to provide an in-depth understanding of participants’ experiences and contexts, shedding light on how they interpret and give meaning to their social environment.^35^ The start of the project was delayed due to the COVID-19 pandemic, as planned in-person observations became unfeasible, prompting a suspension of activities for several months. During this time, the methodology was reformulated to omit observational research and instead conduct online interviews. Following the suspension, field work was initiated in March 2021 and carried out through July 2022.

This article focuses on a sub-sample from the broader study, comprising young women aged 18–25 with diverse sexual orientations. At the time of the interviews, they were residing in municipalities within Chile’s Metropolitan Region, under the administration of the West Metropolitan Health Service. The selected municipalities are classified as high or medium-high priority by the Ministry of Social Development and Family, indicating elevated social vulnerability based on income, education, and health indicators. ^36^

### Participant recruitment and selection

Participants were recruited through purposive and snowball sampling. Given that recruitment took place during the COVID-19 pandemic, they were contacted through virtual means. An open call for research participation was shared on social networks of youth organisations based in the target municipalities. Some of these organisations were previously known to the research team, while others were identified through searches on Facebook and Instagram. Local sexual health teams also helped to disseminate the call through healthcare centres. This strategy generated initial interest, which was expanded through snowball sampling to reach additional contacts.

Initial contact with each participant was made via email, WhatsApp, or social media messages, followed by a phone call to explain study details, participation requirements, and ethical considerations. Participants who expressed interest received the research protocol and consent forms to formally confirm their participation.

The sample analysed in this article comprised 23 participants aged 18–25, as detailed in Table 1. At the time of the interview, all participants lived with family members except three (one who lived alone, and two with friends). Some had previously lived independently, with friends or couples, but had returned to live with family during the COVID-19 pandemic. 20 participants identified themselves as feminists, and three preferred not to use the label. All but two participants were pursuing higher education, in most cases supported by scholarships or financial aid, reflecting a relatively privileged position despite their challenging environment.

**Table 1.** Participant characteristics (*N* = 23)

N°	Pseudonym	Age	Municipality	Gender and sexual identity	Education
1	Cala	22	Quinta Normal	Woman, heterosexual	Technical institute student
2	Muriel	23	Cerro Navia	Woman, heterosexual	University student
3	Paloma	22	Cerro Navia	Woman, heterosexual	University student
4	Amelia	22	Cerro Navia	Woman, heterosexual	University student
5	Camila	22	Pudahuel	Woman, fluid sexuality	University student
6	Marcela	24	Lo Prado	Woman, bisexual	University student
7	Bárbara	23	Quinta Normal	Woman, fluid sexuality	University student
8	Fresia	22	Cerro Navia	Woman, bisexual	University student
9	Kathya	24	Quinta Normal	Woman, heterosexual	University student
10	Daniela	18	Lo Prado	Woman, fluid sexuality	High school student
11	Cloe	19	Pudahuel	Woman, bisexual	Technical institute student
12	Madelein	20	Cerro Navia	Woman, did not want to label her sexuality	Completed high school
13	Bianca	25	Quinta Normal	Woman heterosexual	Graduated from university
14	Jacinta	22	Pudahuel	Woman, heterosexual	University student
15	Irina	22	Pudahuel	Woman, did not want to label her sexuality	University student
16	Gabriela	25	Cerro Navia	Woman, heterosexual	Technical institute student
17	Antonia	22	Pudahuel	Woman, did not want to label her sexuality	University student
18	Josefina	21	Lo Prado	Woman, bisexual	University student
19	Fernanda	24	Cerro Navia	Woman, bisexual	Completed high school
20	Maira	22	Lo Prado	Woman, heterosexual	University student
21	Florencia	22	Cerro Navia	Woman, pansexual	Technical institute student
22	Mariana	23	Curacaví	Woman, bisexual	University student
23	Ana	24	Curacaví	Woman, heterosexual	University student

All participants identified as women at the time of interview. Ten participants identified themselves as heterosexual. Of these, two explained that while they consider themselves heterosexual, they have had or would consider having relationships with women. Six participants identified as bisexual. One identified as pansexual, meaning she is attracted to individuals regardless of their gender or sexual identity. Three participants described themselves as being “fluid,” meaning their sexual orientation or attraction may change over time and is not fixed to a single category. And lastly, three interviewees preferred not to label their sexuality. Table 1 shows the gender and sexual identity of the participants according to these categorisations, which corresponds to the one given by them at the time of the interviews.

### Data collection

In-depth interviews were employed to gather detailed insights into participants’ perceptions and experiences related to SRH care. The interview guides were developed by AO, MS, and CR, drawing from the study objectives and informed by a comprehensive review of current literature, with refinements made throughout the research process. Researchers AO (PhD) and MS (PhD) are anthropologists with extensive experience in qualitative research, including expertise in the SRH of young people and in assessing public policies on these matters.

Questions were open-ended to allow participants the freedom to provide deep, detailed, and unexpected insights into their perceptions and experiences. The main themes covered were: (1) Life narrative with a focus on health and SRH; (2) Representations and manifestations of gender, sexuality, and SRH; (3) Resources in SRH; (4) Relationships and experiences with biomedical health sector regarding SRH.

Initially, most interviews were conducted online, while COVID-19 restrictions were still in place. Later sessions included both virtual and in-person formats, when restrictions were eased. The researchers had initial concerns that online interviews might impede their ability to establish rapport with participants and encourage in-depth responses. However, as the interviews progressed, the richness of the information collected indicated that this was not a significant barrier. The quarantines in Chile and the emergence of new virtual spaces facilitated opportunities for introspection and reflection among participants.

Interviews were conducted by researchers and authors AO, MS, and CR. Each interview commenced with an introduction from the researcher, a review of informed consent, and an opportunity for participants to ask questions or voice any concerns. Recording of the interviews began after these initial interactions were completed. Interviews were conducted in Spanish, lasting 60–90 minutes each. Two participants had a second interview to cover topics not addressed in the first session, ensuring all questions from the interview guide were thoroughly explored.

### Qualitative data analysis

The interviews were recorded and transcribed using Microsoft Word. Transcripts were checked for accuracy using the original recording by the interviewers. Thematic analysis was used to code and analyse the data^37^ using the software ATLAS T18. The coding process enabled a systematic mapping of the data, providing an overview of disparate data and, thereby, allowing a coherent interpretation in relation to the research questions.^38^ All authors conducted this analysis by (1) studying the transcripts, (2) noting emerging issues and topics from the data, (3) identifying preliminary codes, (4) mapping the relationships between different codes, (5) creating a code book with main and minor codes, (6) identifying patterns throughout the data. All completed each step individually, and then reviewed and compared their findings to reach a consensus. CW, a native English speaker, translated the quotes, and all translations were checked by the authors to ensure the original meaning remained.

The study considered triangulation, theoretical–methodological adequacy, and audit trail as criteria of rigorousness.^37,39^ Regarding triangulation, the information analysis was contrasted between the researchers of the team, who have backgrounds in different social sciences and health disciplines: social and medical anthropology (AO, MS, and CR), nursing, midwifery, and social epidemiology (BC), and political science (CW). This helped to secure consistency in the interpretation of the information. In terms of theoretical and methodological rigour, the research team ensured during the study’s design phase that the research problem was logically aligned with the chosen methodology and theoretical framework, maintaining coherence throughout the study. Additionally, the team maintained a consistent audit trail by keeping a detailed field diary to document ideas and experiences. This record complemented the in-depth interviews, contributing to a more robust and trustworthy process of data analysis and interpretation.

### Ethical considerations

The study was approved on 10 October 2019, by the Ethics Committee of Facultad de Medicina Clínica Alemana, Universidad del Desarrollo, which is officially registered with the US Office for Human Research Protections. Participants received detailed information about the study both orally and in writing, including the benefits, potential risks, study procedures, and details regarding audio recording, data storage, and confidentiality. Informed consent forms were signed by all participants before the interviews. To ensure confidentiality and anonymity, participants’ names were replaced with interview codes and pseudonyms, and any personal information that could identify a participant was removed. The data have been securely stored in a database accessible only to researchers directly involved in the study, and audio recordings were deleted after transcription.

A research protocol was created to support participants in case they experienced emotional stress during the research process, including support measures for researchers to implement and provisions for referring participants to a psychologist or other relevant health services if needed. This protocol was not required during the research.

## Results

The results are organised according to three of the main topics covered in the interviews, with each topic encompassing several themes identified through qualitative analysis, as outlined in Table 2. These themes construct a narrative that highlights the key factors shaping the relationships between young women, their sexual health, the biomedical health sector and other resources to access SRH support and care.

**Table 2.** Topics and identified themes

Topics	Description	Themes
New scenarios in young women’s sexualities	Examines how young women perceive, interpret and express their sexuality within the context of the local socio-political background	The lived body Diverse identities beyond binary classifications Self-care, self-exploration, pleasure, and emotional responsibility
SRH in the formal health system	Describes young women’s perceptions of SRH care in the official biomedical system	Fragmented and traditional representations of the body and sexuality Over-medicalisation Positive experiences of care
Diversification of SRH resources	Describes the diverse resources that young women utilise to access the SRH support and care they require	Strategic use of biomedical SRH services Health professionals outside of traditional institutions and civil society organisations

### New scenarios in young women’s sexualities

#### The lived body

When referring to their embodied experiences, the interviewees describe integral bodies in which the biological, emotional, and even political dimensions are intertwined. The concept of integrality is central to their testimonies: Bianca (25, heterosexual) defines sexuality as “*not just the sexual act but something much deeper, which encompasses the emotional and psychological aspects, how we relate to others and define and identify ourselves”*. Gabriela (25, heterosexual) adds that “*it covers many dimensions including the emotional and even spiritual, and many sexual forms and practices, beyond the conventional ones”*. Camila (22, fluid) views sexuality as “*a broad spectrum that encompasses sex itself, gender identity, eroticism … it encompasses various aspects of life, whether interacting with others or with oneself”*. In this context, several participants describe their bodies as relational, initially constructed within themselves and later with others. These bodies are characterised by the integration of their lived experiences, their positions within the social fabric, and the violence and care they have encountered. Some of the interviewees explicitly replace the Spanish word “cuerpo” (body), to the feminine counterpart “cuerpa”, to draw attention to a distinctly feminist body. For instance, Marcela (24, bisexual) states:

*“This approach of the ‘cuerpas’ implies that all ‘cuerpas’ have the same rights, it is about us demanding that our bodies are not violated”.*

The participants were high school or early university students in 2018, during Chile’s feminist student mobilisation. This widespread movement had a profound impact on their lives, with interviewees highlighting how it shed light on deeply entrenched gender inequalities that had long been normalised in society:

“*The feminist movement made me realise a lot of things that I saw as normal, which were not normal. It was normal for me to walk home feeling afraid, to be told ‘You have to watch out for yourself’, and ‘You can’t wear these clothes’. I realised that it wasn’t right … I had to deconstruct myself. (Antonia, 22, no label)*”

Additionally, the participants highlight the importance of building a community of peers who share the experience of enduring various forms of gender-based violence and discrimination: “*There were many topics that were not discussed, yet today are valid topics of conversation, and you can confront them with your friends”*(Paloma, 22, heterosexual), “*I understood that it wasn’t just me who suffered these types of violence, but that we were many”* (Muriel, 23, heterosexual).

For these reasons, the participants believe the feminist movement has sparked profound, cross-generational transformations: “*Feminism has brought a lot of change … we can see it in the older generations, they have also changed their perceptions through the movement”* (Josefina, 21, bisexual).

Following the 2018 mobilisation, momentum continued as nationwide protests erupted in the 2019 social uprising, which highlighted deep-rooted socioeconomic inequities across all areas of social life. Eight of the interviewees were actively engaged in these movements, while most others participated less directly or followed the movements through media and social networks.

Coming of age during these turbulent political times profoundly influenced participants’ perceptions of their sexuality and bodies, prompting them to question and, in some cases, resist the historically conservative norms around sexuality and gender embedded in the country’s institutions and families. In this context, none of the interviewees reported having received satisfactory sexuality education from either their schools or families. By the time of the interviews, most participants described undergoing a journey of self-discovery and peer learning about embracing and understanding their sexualities. This process involved a shift from feelings of guilt and fear to a growing sense of acceptance and enjoyment of their sexuality. For example, Josefina (21, bisexual) reflects on growing up in a religious household, stating: “*When I began experiencing pleasure, the guilt began, and from that point on, my body was pure shame*”. Paloma (22, heterosexual), who experienced sexual abuse at a young age and grew under her mother’s descriptions of sexuality as “prohibited and dangerous”, felt profound guilt about her early sexual encounters. However, through self-education and support from social networks and peers, she embraces “a fuller sexuality”.

Participants’ journeys of self-discovery and sexual exploration were not without obstacles, particularly during the COVID-19 pandemic. Many were beginning to explore their sexuality with partners, grappling with numerous questions about their sexual health and seeking support on the topic, yet encountering significant barriers to accessing in-person health services. Jacinta (22, heterosexual) highlighted the challenges she faced in understanding and managing her menstrual cycle during the pandemic.

Irina expressed her disappointment over the shift from in-person consultations with an SRH team to phone appointments for her contraceptive prescription. She also shared how she supported a friend seeking an induced abortion – illegal in the country – by turning to online peer networks for assistance:

“*We looked for webpages and groups to help in these cases. She contacted an online abortion line and they made a meeting via Zoom, and it was a very complex period because there was not much access to the pills*.” (Irina, 22 no label)

Cala (22, heterosexual) described her experience of experimenting with contraceptives during the height of COVID-19, following a particularly difficult period of mental health that she linked to the contraceptive pill. These comments indicate that young women experienced significant harm during the pandemic due to reduced access to SRH care, leaving them to educate themselves instead.

#### Diverse identities beyond binary classifications

The participants’ personal experiences and perceptions reflect broader societal shifts in understandings of gender and sexual identities. They describe ongoing transformations in how they engage with these issues, shaped significantly by the socio-political context and their interactions with social networks and peers, as Bárbara describes:

“*It was only with the feminist revolt that I opened my eyes and it became visible that gender is not only female and male. I began to understand as well that I am not a woman, and I do not identify with that gender. But no one asks you that as a girl*.” (Bárbara, 23, fluid)

Across the sample, there was a shared understanding that individuals should have the freedom to explore their sexual and emotional preferences. Among the 10 participants who identified as heterosexual, most expressed comfort with this identity, while two indicated openness to exploring same-sex relationships, highlighting that they do not view their sexual identity as fixed. This more fluid understanding of sexuality was common across participants, who embodied a broad spectrum of expressions that resist static categorisation. Notably, three participants explicitly described their sexual orientation as “fluid”.

“*If you asked me if I am attracted to women or men, I think both because in reality it is not like I am attracted to sex but to the person, so I don’t have a definite sexual orientation.*” (Daniela, 22, fluid)

Three other participants chose not to label their sexuality, emphasising its dynamic and evolving nature. As Madelein (20, no label) explained: “*If someone asks me if I’m bisexual, for me it’s like: ‘I don’t know, I like people’”*. Yet others did identify with a non-heterosexual label, even while acknowledging it could shift over time. For instance, Josefina explains:

“*All my life, I thought I was heterosexual, yet now I’m not sure if that’s how I would define myself. I am attracted to men, but I do not feel comfortable around them. I have always thought I like people in general, regardless of whether they are men or women*.” (Josefina, 21, bisexual)

These diverse identities often clash with conservative views in Chilean society, and some participants choose to avoid these topics with their families. Fernanda (24, bisexual) shares: “*I had a relationship with a woman – it was a nice, affectionate relationship. My family doesn’t know, obviously, but my friends had no issues with it*”. Antonia (22, no label) similarly notes: *“My dad assumes I’m straight, so why should I have to make him understand that maybe I’m not?”*

#### Self-care, self-exploration, pleasure and emotional responsibility

All participants emphasised that they had to self-educate on essential aspects of sexuality not addressed by the health system. This includes understanding and caring for bodily cycles, such as menstruation, which all participants mentioned as integral to their experience of sexuality. As Kathya expresses:

“*Sexuality also begins with taking care of the body. For instance, menstruation is part of sexuality – it’s a huge topic that nobody teaches you about. It’s something you have to learn on your own: how to manage physical and mental symptoms, what products to use, what alternatives exist, and their effects*.” (Kathya, 24, heterosexual)

Self-care in sexuality entails paying attention to bodily cycles and prioritising self-exploration, including masturbation. As Cala (22, heterosexual) put it:

*“Masturbation is a fundamental part of my life … I think it’s very positive and greatly helps relationships – knowing yourself, what you like, and understanding your limits”.*

Mariana (23, bisexual) shares:

*“When you know yourself and understand what gives you pleasure, you can experience much more open and pleasurable sex”.*

Pleasure is highlighted as a central aspect of sexuality, as Kathya (24, heterosexual) notes: “*Sexuality is closely tied to pleasure and not only the sexual pleasure of an orgasm or climax but also to an overall sense of well-being*”. Participants agree that pleasure has often been overlooked in traditional sexual health narratives. Cloe expresses:

“*People only thought about men, women seemed to be left out, as if they didn’t need pleasure. I had never been given the options that there are to have pleasure. But now I feel much more confident, given that the same sources that taught me about feminism talk about women’s pleasure*.” (Cloe, 19, bisexual)

Another dimension of self-care highlighted in various testimonies is the importance of understanding one’s “sexual biography” for cultivating healthy sexuality. Participants often refer to “integrating”, “working on” or “healing” from experiences of sexual abuse and harassment – which several of the participants had experienced – which profoundly impact their relationship with their own sexuality. Maira (22, heterosexual) reflects: “*I’m still on the path to … it’s still difficult in terms of how my body reacts. I’m unsure if this is linked to past abuse, so I’m working on it*”. These experiences of sexual violence are woven into their embodied biographies, and participants regard the act of sharing these narratives as a meaningful practice of care. Fresia (22, bisexual) notes: “*Conversations about violence lead us to a sense of sexual responsibility and greater awareness, fostering a deeper sexual consciousness and connection with others”*.

Participants view a positive relationship with one’s body and sexuality as foundational for respectful and pleasurable sexual interactions with others – emphasising consent, mutual pleasure, “emotional” or “affective responsibility” as key components of healthy relationships. As Jacinta expresses:

“*I think we have learned, collectively, to recognise red flags and to understand emotional responsibility, ensuring that relationships are honest and respectful of boundaries*.” (Jacinta, 22, heterosexual)

### Sexual and reproductive health care in the formal health system

#### Fragmented and traditional representations of the body and sexuality

In stark contrast to the comprehensive views of sexuality articulated by the participants, they agree that the health system predominantly promotes fragmented representations of the body and sexuality. Their experiences with SRH care in both the public and private sectors often involve more paradigmatic mismatches than meaningful interactions. Care tends to focus on the physiological body, often undermining the emotional dimensions of care, as expressed by the participants. Many recall past experiences in the formal healthcare sector that left them feeling uncomfortable, fearful, or ashamed. Mariana (23, bisexual) states she will not return to the formal health sector after enduring several negative experiences, such as being prescribed contraceptive pills without any prior discussion or examination or being scolded when expressing that a vaginal examination was painful.

The participants express that the medical system is ill-equipped to address various forms of diversity. Camila, a midwifery student at the time of the interview, states:

“*We are only trained to deal with urban white women who can read and write and have a positive relationship with their bodies. I lack the tools to engage with adolescents or adults who are drug-dependent or in vulnerable situations. I also don’t have the resources to address multiculturalism, whether it pertains to indigenous people or migrants. Additionally, I’m not equipped to work with transgender individuals. Most importantly, I feel least prepared to assist people who are incarcerated, homeless, or experiencing psychological disorders. There is a clear absence of a biopsychosocial approach*.” (Camila, 22, fluid)

A key gap identified by participants is the lack of tools within the healthcare system to adequately address sexual diversity. Many participants, particularly those who do not identify as heterosexual, report feeling misunderstood or unheard regarding their identities and sexual practices during interactions with healthcare providers. In most cases, they describe healthcare professionals assuming heterosexuality, which undermines the relevance and quality of care they receive. While some participants actively disclose their sexual-affective identities, others choose not to, often resulting in feeling overlooked or inadequately attended to. Fernanda (24, bisexual) articulates this concern: “*They only think of sexuality as penetration between heterosexual partners, but it goes way beyond that*”. This heteronormative focus is evident in the prioritisation of contraception, implicitly assuming pregnancy risk as the central concern. As Marcela (24, bisexual) observes: “*Young people find little value in what exists [in the formal healthcare sector] today. The health system is not designed to support diverse identities related to culture, socioeconomic status, gender identity, or sexual diversity*”.

#### Over-medicalisation

A significant theme that emerged was some participants’ critique of what they perceive as the over-medicalisation of their SRH. Several recalled being prescribed contraceptive pills following menarche for issues such as heavy bleeding, menstrual pain, polycystic ovary syndrome, acne and weight management. As Josefina (21, bisexual) noted, “*The midwife told me my weight gain was probably due to a hormonal disorder and prescribed me contraceptive pills*”, yet no other explanations or options were explored – a pattern shared by other participants.

This recommendation is made without considering their sexual identities, typically assuming they are having sex with men, neglecting to discuss potential side effects, or presenting alternative options. Camila explains:

“*When I went for my first smear test, I was told that I needed to focus on preventing pregnancy, and that the only option, and the best thing I could do for myself, was the pill because it regulated my cycle and everything else. I said ‘yes’ because I didn’t know much about the topic*.” (Camila, 22, fluid)

Ana critiques the medical system for prescribing hormonal contraceptives to young girls, sharing her experience:

“*They don’t truly care for you; they simply give you a pill without considering whether you get sick or feel unwell. (…) You have to accept that you might gain weight, develop acne, experience swelling in your legs, and endure the long list of side effects that come with contraceptive pills for women*.” (Ana, 24, heterosexual)

Several participants shared negative experiences with hormonal contraception, highlighting the tensions between their lived bodies and the medicalisation of their sexual health. Cala (22, heterosexual) stopped taking the pill after linking it to her anxiety and depression. Muriel (23, heterosexual) similarly decided to stop taking the pills, stating: “*I had suffered a lot and didn’t want my body to go through that again. I explained to my partner that they [the pills] were hurting me and making me feel very bad*”.

#### Positive experiences of care

Amidst these challenges, participants also shared positive experiences within the formal health system, recounting encounters where they felt genuinely respected and treated in a comprehensive way. For instance, Madelein (20, no label) praised the public sector: “*Everything is very organised, and everyone is very kind and attentive*”*.* Gabriela (25, heterosexual) recalls with “tenderness” her first appointment with a midwife in the public system at age 13. After a non-penetrative sexual encounter, she feared she might be pregnant. The midwife took the time to talk to her about sexuality: “*She gave me a mini talk I had never received before – it was a very meaningful experience*”. Camila (22, fluid), after negative experiences in the public system, switched to private care, where she found a gynaecologist who took an integral approach to her health: “*She explained how hormones can have a big impact on the body, so if I wasn’t feeling well, we would conduct a full evaluation to see what was going on*”*.* Similarly, Daniela found in private care a provider who:

“*Listened to my entire history, asked about my family, and it was clear she was paying attention. Everything was tailored to what I shared - it felt very personal, very specific to me*.” (Daniela, 18, fluid)

Most participants, however, described these encounters as “exceptions” or felt they had simply “been lucky”, suggesting that such positive experiences are not the norm. As Irina put it:

“*I am fortunate to have a great gynecologist. She is very knowledgeable and takes the time to explain everything. She provides all the options and reassures you when you’re feeling scared. She asks how you’ve been and really listens*.” (Irina, 22, no label)

While these kinds of positive experiences do occur within the formal healthcare system, several participants declared finding more consistent and affirming care outside of it, as will be discussed in the following section.

### Diversification of SRH resources

#### Strategic use of biomedical SRH services

Participants report strategically navigating the official healthcare services, fully aware of their limitations. They might approach the system primarily to obtain specific services, such as contraceptive methods, condoms, or STI and Pap tests, while utilising other resources to answer more complex needs. While all participants are insured under the public health system, many transition between public and private providers, utilising private services when affordable to better address their specific needs. For example, Maira (22, heterosexual) is motivated by a desire for more choices in her healthcare, driven by a lack of trust in the public system due to previous experiences with pills that caused her hypertension. Similarly, Camila (22, fluid) sought private specialists because of long wait times in the public system and felt that public healthcare professionals did not adequately address her needs.

The limited approaches of formal institutions toward sexuality prompted participants to use a range of resources to address their SRH needs, with online resources playing a key role in shaping their therapeutic pathways. While this trend existed before COVID-19, it intensified during the pandemic, as many participants sought alternative means to replace the care they had previously received face-to-face from the official health system.

For many participants, the internet has become a key – if not primary – source of information and guidance for managing their SRH. Jacinta (22, heterosexual) shares: “*I learned alone, searching the internet*”*.* Amelia (22, heterosexual) notes: “*I search on Google for information about my infections, I don’t see healthcare professionals*”. Josefina (21, bisexual) highlights the role of social media, particularly TikTok, as valuable resources*:* “*[On TikTok] they discuss a lot of topics like sexology, reproduction, and pleasure … I follow those pages because they explain things in a very natural way*”.

Participants recognise the risks of encountering misinformation online and take steps to mitigate these dangers by consulting reliable sources, such as accredited health professionals or reputable institutions. Other resources to seek for information, counselling and care are discussed below.

#### Health professionals outside of traditional institutions and civil society organisations

Among the most frequently mentioned online resources, many participants highlight engaging with biomedical professionals outside traditional institutions, often seeking advice from their social media pages, either individually or through civil society organisations with which they are affiliated. Participants note that, especially since the onset of the pandemic, there has been an increase in professionals and civil society organisations working to address gaps in SRH care left by formal sectors. These are predominantly young professionals and activists who strive to make SRH care more accessible through holistic, gender-sensitive, and rights-based approaches. Interviewees widely agree that these resources provide valuable guidance.

For example, Florencia (22, pansexual) follows numerous feminist activism pages and feminist gynaecologists on Instagram: “*Gynecologists and midwives on Instagram talk openly about issues related to sex and sexuality, which is very rarely the case when you visit a professional in person*”*.* Camila (22, fluid) adds: “*The landscape has changed a lot, I follow the association of feminist gynecologists, and it’s a much more open approach to sexuality*”*.* Bianca agrees:

“*Today, everything is on Instagram, it’s a great source of information. I follow sexologists teaching sexual education and psychologists offering advice on emotional responsibility, among other topics, and it's wonderful*.” (Bianca, 25, heterosexual)

Another highly valued element of these organisations is their ability to build networks. On one hand, they facilitate connections among people facing similar life situations, fostering mutual support in their respective journeys. On the other, they enable individuals to contact experts quickly and, in many cases, free of charge. Participants further mention that on numerous occasions, these professionals and organisations have stepped in to fill gaps left by the formal healthcare sector, providing online prescriptions or counselling when official healthcare services are inaccessible. While this support initially surged during exceptional circumstances, such as the 2019 social uprising or the COVID-19 pandemic, it has since extended over time, continuing to address structural gaps in the healthcare system.

Some participants highlighted support networks that help individuals access information and assistance for self-managed abortions, particularly in response to the country’s highly restrictive abortion laws, which exclude most unplanned pregnancies. These organisations provide guidance on medication use and offer emotional and practical support throughout the process. Three participants describe their experiences, highlighting the crucial role these networks play in navigating such stressful situations. Bianca shares:

“*First, I had to buy the pills on the black market, but it didn’t work because I took too small a dose. Then I learned I needed to do it in a safer way, so I connected with an organisation. With their help, I was able to terminate the pregnancy successfully. Since then, I’ve also supported other women in their abortion processes.*” (Bianca, 25, heterosexual)

Gabriela (25, heterosexual) also reached out to an organisation for guidance, though ultimately, she was able to independently obtain the pills she needed more quickly by purchasing them through mail order from abroad. Irina (22, no label) recalls supporting a friend through the process: “*We searched for websites and support groups and found an abortion hotline. They explained everything in detail and were very helpful*”.

## Discussion

The findings of the study reveal persistent gaps between the holistic ways in which young women conceptualise their bodies and sexuality, and the dominant frameworks guiding SRH within the formal healthcare system. A conservative social framework lacking transversal approaches to comprehensive sexual education is compounded by a biomedical system characterised as reductionist, adult-centred, risk-oriented, and heteronormative. Although these deficiencies have been documented in the country for over a decade,^11,24,40,41^ recent socio-political movements – the feminist movement of 2018 and the social unrest of 2019 – have brought a new lens to these longstanding issues. This is especially true for youth, who had a leading role in these movements, as evidenced by data from the most recent National Youth Survey in 2022^25^: young people aged 15–29 reported the highest level of political interest in a decade (29%); over half of the young population (57.3%) reported engaging in at least one activity during the social uprising – almost 20% more than adults; and 54% declared actively participating in at least one social organisation (double of the reports from 2018). Their participation enabled and encouraged a novel questioning of entrenched structures, with gender norms at the centre of discussion.

In Chile, these social movements, especially feminism, have exposed deeply ingrained sexism and normalised violence while fostering a sense of community and peer support among young women. This is shared with other Latin American countries, where recent feminist movements have increasingly embraced diversity, promoting multiple identities that engage in dialogue and challenge traditional norms. These movements push back against the enduring influence of Catholic morality on secular legislation, advocating for more inclusive and plural understandings of sexuality and rights.^42,43^

As the interviewees reveal, their experiences were no longer invisible, isolated, or individual. They could connect and share experiences around their “cuerpas”, a linguistic re-signification that sparks a discussion about the patriarchal colonisation of bodies, challenging disciplinary and control logics often imposed on female bodies and LGBTIQA+ people.^44^ The “cuerpa” embodies a holistic approach, prioritising self-care, self-exploration, pleasure, and emotional responsibility. These themes are crucial since, as international literature reports, issues like pleasure are often left out of sexual and reproductive health and rights (SRHR) programmes and interventions.^45^

Moreover, the COVID-19 pandemic posed significant challenges to SRH care for young people, disrupting in-person services, access to prevention methods, and education, while social isolation and economic insecurity led many – like several of our interviewees – to move back in with their families. This shift significantly impacted their sexual and emotional relationships.^46,47^ The pandemic prompted a shift toward seeking alternative resources to in-person health services, primarily through the internet. For some participants, this transition helped bridge gaps in access to health-related information and education by offering timely, accurate, and personalised responses to their health questions, as observed in other contexts.^48^ It also encouraged young women – heterosexual and non-heterosexual – to create online networks, engage in discussions about SRH with peers, and find support from health professionals and civil society organisations accessible online. These networks often provided better responses to their needs than the formal healthcare system, as they embraced comprehensive approaches to sexuality and addressed the needs of sexually and gender-diverse populations. This phenomenon is not unique to Chile and has been reported in various global contexts.^49,50^ Thus the internet and social media have become central channels for seeking sexual health information among young people, particularly LGBTIQA + youth by playing a critical role in accessing information and sharing experiences related to sexual health.^51^

All of the above aligns with the integrated definition of SRH and rights proposed by the Guttmacher-Lancet Commission in 2018, which frames SRH as a state of physical, emotional, mental, and social well-being in all aspects of sexuality and reproduction, and not merely the absence of disease, dysfunction, or infirmity. The Commission advocates for a positive approach to sexuality and reproduction, recognising the role of pleasurable sexual relationships, trust, and communication in fostering self-esteem and overall well-being. Additionally, it asserts that everyone has the right to make decisions about their bodies and to access services that support those rights.^52^ This positive approach is precisely what young women are seeking outside the official healthcare system. It is particularly important for non-heterosexual women, who often face homophobia and heteronormative assumptions in healthcare settings – factors that can delay diagnosis, hinder treatment, and create barriers to accessing services.^53,54^ In Latin America, for instance, the presumption of heterosexuality in gynaecological care tends to render female homoerotic practices invisible, as shown in a recent study in Chile which found that 91% of survey respondents reported that health professionals assumed them to be heterosexual during SRH consultations.^55^ As a result, many non-heterosexual women report avoiding medical visits due to experiences of stigma and discrimination.^56^

Our findings lead to several recommendations that could help strengthen the provision of SRH services within the formal healthcare system, while also leveraging existing resources, networks, and organisations. Young women express a comprehensive approach to SRH, in which sexuality is integrated into a broader conception of health, including affectivity, social relationships, mental health, and experiences of violence. Greater integration between SRH, mental health, and gender-based violence services is essential to ensure equitable and responsive care. The “3A” program in Lo Prado, Chile, offers a promising example of comprehensive adolescent health education delivered through multidisciplinary collaboration.^57^ Although currently limited to some schools, its model could be expanded across Youth-Friendly Health Services for adolescents and serve as a valuable reference for the design of programs aimed at young adults.

In line with young women’s need to be treated in more comprehensive terms, it is also important to acknowledge the critique some raise regarding the over-medicalisation of their SRH, particularly the routine prescription of hormonal contraceptives. Their concerns relate to adverse physical and emotional side effects, insufficient information about non-pharmacological alternatives, and limited involvement in decision-making. This critique signals a broader demand for more holistic approaches to SRH. In response, the formal healthcare system should adopt more flexible and inclusive models that acknowledge diverse experiences and preferences, ensuring that young women are informed, respected, and actively engaged in shaping their care pathways.^58^

It is also essential to ensure a non-heteronormative approach to care, one in which women feel safe to disclose their identities and practices to receive care that is tailored to their specific needs. This requires improved training for healthcare professionals on these issues and could be further strengthened through collaboration with – or even integration of – civil society organisations into the healthcare network. These organisations, which have become trusted resources for young people, bring valuable expertise. While historically many have focused on gay men and HIV, a growing number now include or specifically focus on women. This leads us to reflect on the potential impact of strengthening collaborative ties between the formal healthcare system and youth-led civil society organisations, which aligns with the World Health Organization’s^59^ strategies for advancing STI prevention, which emphasise the need to integrate service delivery components in innovative ways, coordinating efforts both within and beyond the health sector. One of WHO’s five strategic directions is to “engage civil society and empowered communities”, which includes encouraging key and affected populations to take leadership roles in service delivery, advocacy, and policy development to ensure services are culturally appropriate and responsive to community needs, while addressing stigma, discrimination, and structural barriers.^59^ The role of non-governmental actors has thus become central to contemporary debates on strengthening governance for global health – that is, the institutional arrangements that facilitate collective action to address shared health needs.^60^

Implementing effective community engagement strategies that enable young people, particularly young women, to participate in the design and delivery of tailored health services is also essential. Globally, meaningful community involvement has been shown to positively influence the advancement of sexual and reproductive rights.^61^

This study represents an unprecedented effort in Chile, shedding light on the perceptions, practices, and needs of young women in SRHR. Its findings provide a foundation for informing the design of public policies at the national level and across Latin America and the Caribbean.

In terms of limitations, the recruitment approach may have introduced selection bias, as it relied on participants’ digital connectivity and online presence. Consequently, the sample likely over-represents young women with reliable internet access, digital literacy, and some degree of organisational engagement, potentially excluding those who are more socially or digitally isolated. The lack of in-person outreach, due to pandemic restrictions, further limited access to young women who do not frequent health centres or participate in youth networks. These factors restrict the generalisability of the findings to similarly urban, connected, and engaged populations. To mitigate this, we deliberately sought heterogeneity in later recruitment – considering socioeconomic background, education level, organisational involvement, and sexual orientation – to broaden the range of perspectives captured within the study’s constraints.

Additionally, the qualitative nature of the data means the insights reflect the experiences of a specific group of young women from Chile’s Metropolitan Region, whose shared characteristics limit wider generalisability. Although all participants had grown up and were living in high-vulnerability neighbourhoods, nearly all were pursuing or had completed university or technical education, placing them at a relatively higher educational level within their context. Moreover, most identified as feminists, which may have positioned them to be more critical of heteronormative and patriarchal structures than the general population.

Future research should seek to include a more diverse sample, particularly in terms of education, geographic location, and ideological orientation, to better capture the varied experiences of young women across different social contexts. The study also did not encompass the full spectrum of gender and sexual identities, which highlights the need for future research to explicitly include these groups. Finally, conducting interviews online due to the pandemic presented some challenges, such as limited in-person rapport. However, we consider this limitation minor, as participants remained open and engaged, and the format allowed for thorough exploration of all topics. This aligns with recent literature showing that online methods can foster safe, non-judgmental environments that enhance comfort and trust, especially among marginalised or underrepresented groups.^62^ Despite these limitations, the study provides valuable insights into key aspects of SRH and rights among young women in contemporary Chile.

## Conclusion

This study reveals that for many young women, particularly those who identify as feminists, articulate diverse sexual and affective orientations, and have been engaged in recent socio-political mobilisations, current health system frameworks appear insufficient, shaped by reductionist, fragmented, heteronormative and risk-centred logics that fail to address the complexity of their lived realities. These young women articulate an understanding of sexuality that is holistic, relational, and grounded in notions of self-care, pleasure and emotional responsibility. Although gaps in access and quality have been documented in the literature, they have become increasingly visible in the wake of recent socio-political movements, and further amplified with the COVID-19 pandemic, which have challenged traditional conceptions of gender and health.

In this context, new organisations and platforms emerged to fill these gaps, often offering more accessible, inclusive, and comprehensive support – ultimately accelerating a broader shift toward finding SRH resources outside the formal system, which in many cases better align with young women’s needs and expectations. Such a shift could be supported through strengthening collaboration with, or integrating, alternative health resources – such as those offered by civil society organisations – into the formal healthcare network, thereby reinforcing and expanding the progress already made in SRH within the health system. While this study offers valuable insights into the experiences of a specific group of young women, future research should aim to include more diverse social, geographic, and educational backgrounds to broaden understanding and strengthen the applicability of findings.

## Data Availability

The data supporting the findings of this study are available upon request from the corresponding author, MS. These data are not publicly available as they contain information that could compromise the privacy and consent of research participants.

## References

[1] WHO. The global strategy for women’s and children’s health (2016–2030); 2018. https://www.who.int/publications/i/item/A71-19. 10.2471/BLT.16.174714 PMC4850547 27147756

[2] Correa Urquiza M, Martínez Hernáez Á, Martorell-Poveda MA. Mundos vitales y malestar emocional en el tránsito adolescente: una mirada desde la antropología fenomenológica. In: D Cruz, E Mollejo, F González, editors. Adolescencias. Nuevos Retos, Nuevas Transiciones. Madrid, España: Asociación Española de Neuropsíquiatría; 2021. 69–79.

[3] Mazur A, Brindis CD, Decker MJ. Assessing youth-friendly sexual and reproductive health services: A systematic review. BMC Health Serv Res. 2018;18(1):216. doi:10.1186/s12913-018-2982-4 PMC5870922 29587727

[4] Liang M, Simelane S, Fortuny Fillo G, et al. The state of adolescent sexual and reproductive health. J Adoles Health. 2019;65(6S):S3–S15. doi:10.1016/j.jadohealth.2019.09.015 31761002

[5] Barral R, Kelley MA, Harrison ME, et al. Dismantling inequities in adolescent and young adult health through a sexual and reproductive health justice approach. Semin Reprod Med. 2022;40(1–02):131–145. doi:10.1055/s-0042-1742347 35052004

[6] Brown E, Monaco SL, O’donoghue B, et al. Improving the sexual health of young people (Under 25) in high-risk populations: a systematic review of behavioural and psychosocial interventions. Int J Environ Res Public Health. 2021;18(17):9063. doi:10.3390/ijerph18179063 34501652 PMC8430747

[7] Shaw D. Access to sexual and reproductive health for young people: bridging the disconnect between rights and reality. Int J Gynecol Obstet. 2009;106(2):132–136. doi:10.1016/j.ijgo.2009.03.025 19535075

[8] Barroso C. Beyond Cairo: sexual and reproductive rights of young people in the new development agenda. Glob Public Health. 2014;9(6):639–645. doi:10.1080/17441692.2014.917198 24922347

[9] Obach A, Sadler M, Jofré N. Salud sexual y reproductiva de adolescentes en Chile: El rol de la educación sexual. Rev Salud Publica (Bogota). 2017;19(6):848–854. doi:10.15446/rsap.V19n6.70023 30183841

[10] Gómez- Sánchez P-I. Personal reflections 25 years after the international conference on population and development in Cairo. Rev Colomb Enferm. 2019;18(3):e012. doi:10.18270/rce.v18i3.2659

[11] Macintyre AKJ, Montero Vega AR, Sagbakken M. From disease to desire, pleasure to the pill: A qualitative study of adolescent learning about sexual health and sexuality in Chile health behavior, health promotion and society. BMC Publ Health. 2015;15:945. doi:10.1186/s12889-015-2253-9 PMC4580294 26399632

[12] Morgan L, Roberts E. Reproductive governance in Latin America. Anthropol Med. 2012;19(2):241–254. doi:10.1080/13648470.2012.675046 22889430

[13] UNAIDS. Advancing towards 2020: progress in Latin America and the Caribbean; 2020.

[14] Caffe S, Plesons M, Camacho AV, et al. Looking back and moving forward: can we accelerate progress on adolescent pregnancy in the Americas? Reprod Health. 2017;14(1):83. doi:10.1186/s12978-017-0345-y 28705166 PMC5512880

[15] Cevallos Mendoza M, Moreira A, Burga S, et al. Review on adolescent pregnancy and social implications. Rev Facul Med Humana. 2024;24(2):156–165. doi:10.25176/RFMH.v24i2.6207

[16] Ministerio de Salud. Política Nacional de Salud de Adolescentes y Jóvenes 2008–2015; 2008. http://web.minsal.cl/wp-content/uploads/2015/09.

[17] Rodríguez J, Roberts A. El descenso de la fecundidad adolescente en Chile: antecedentes, magnitud, determinantes y desigualdades. Serie de Estudios N° 12; 2020.

[18] Blamey R, Sciaraffia A, Piñera C, et al. VIH/SIDA Situación epidemiológica de VIH a nivel global y nacional: Puesta al día Epidemiological situation of HIV at global and national level: update. Rev Chilena Infectol. 2024;41(2):248–258. doi:10.4067/s0716-10182024000200248

[19] UNAIDS. The path that ends AIDS: UNAIDS Global AIDS Update 2023; 2023. https://www.unaids.org/en/resources/documents/2023/global-aids-update-2023.

[20] Cáceres-Burton K. Report: epidemiological situation of sexually transmitted infections in Chile, 2017. Rev Chilena Infectol. 2019;36(2):221–233. doi:10.4067/S0716-10182019000200221 31344158

[21] Mondschein S, Quinteros M, Yankovic N. Gender bias in the Chilean public health system: Do we all wait the same? PLoS One. 2020;15(9):e0239445. doi:10.1371/journal.pone.0239445 PMC7515657 32971530

[22] Poblete-Inostroza RN. Comprehensive sexual education in Chile: barriers and opportunities to implement educational programmes. Qualit Res Educ. 2024;13(1):43–63. doi:10.17583/qre.12524

[23] Krauskopf D. LOS MARCADORES DE JUVENTUD: LA COMPLEJIDAD DE LAS EDADES. Última Década. 2015;23:115–128. doi:10.4067/S0718-22362015000100006

[24] Obach A, Sadler M, Cabieses B. Intersectoral strategies between health and education for preventing adolescent pregnancy in Chile: findings from a qualitative study. Health Expect. 2019;22(2):183–192. doi:10.1111/hex.12840 30369026 PMC6433321

[25] INJUV. Décima Encuesta Nacional de Juventudes. Santiago, Chile: Instituto Nacional de la Juventud; 2022.

[26] Green Rioja RA. Collective trauma, feminism and the threads of popular power: a personal and political account of Chile’s 2019 social awakening. Rad Am. 2021;6(1):2. doi:10.14324/111.444.ra.2021.v6.1.002

[27] Obach A, Sadler M, Carreño A. Corporalidades, Sexualidades y Juventudes en un Chile Diverso. Santiago, Chile: Universidad del Desarrollo; 2023.

[28] Paschen-Wolff MM, Greene MZ, Hughes TL. Sexual minority women’s sexual and reproductive health literacy: A qualitative descriptive study. Health Educ Behav. 2020;47(5):728–739. doi:10.1177/1090198120925747 32506954 PMC7568910

[29] Khozah MY, Nunu WN. Sexual and gender minorities inclusion and uptake of sexual and reproductive health services: A scoping review of literature. Am J Men’s Health. 2023;17(4):1–15. doi:10.1177/15579883231184078 PMC10328172 37395415

[30] Tabaac AR, Sutter ME, Haneuse S, et al. The interaction of sexual orientation and provider–patient communication on sexual and reproductive health in a sample of U.S. women of diverse sexual orientations. Patient Educ Couns. 2022;105(2):466–473. doi:10.1016/j.pec.2021.05.022 34023174 PMC8594287

[31] Carreño-Calderón A, Obach A, Correa-Matus E. “I hadn’t known a midwife until I got pregnant”: sexual and reproductive healthcare for Mapuche youth women, Chile. Saude e Sociedade. 2024;24(1):e230520es. doi:10.1590/S0104-12902024230520es

[32] Obach A, Blukacz A, Sadler M, et al. Barriers and facilitators to access sexual and reproductive health services among young migrants in Tarapacá, Chile: a qualitative study. BMC Publ Health. 2024;24(386). doi:10.1186/s12889-024-17884-5 PMC10845769 38317103

[33] Obach A, Sadler M, Roberts A, et al. The role of new civil society organizations in promoting sexual health in Chile. Rev Antropol Soc. 2023b;32(2):169–184. doi:10.5209/raso.91748

[34] Tong A, Sainsbury P, Craig J. Consolidated criteria for reporting qualitative research (COREQ): a 32-item checklist for interviews and focus groups. Int J Qual Health Care. 2007;19(6):349–357. doi:10.1093/intqhc/mzm042 17872937

[35] Patton M. Qualitative research & evaluation methods. Thousand Oaks, CA: SAGE Publications; 2022.

[36] Ministerio de Desarrollo Social y Familia, & Seremi Regional Metropolitana. ÍNDICE DE PRIORIDAD SOCIAL DE COMUNAS 2022; 2022.

[37] Vásquez Navarrete ML, Ferreira da Silva MR, Mogollón Pérez A, et al. INTRODUCCIÓN A LAS TÉCNICAS CUALITATIVAS DE INVESTIGACIÓN APLICADAS EN SALUD. Programa Editorial Universidad del Valle; 2006. doi:10.25100/peu.188.

[38] Elliott V. Thinking about the coding process in qualitative data analysis. Qualit. Report. 2018;23(11):2850–2861. doi:10.46743/2160-3715/2018.3560

[39] Hernández Sampieri R, Fernández Collado C, Baptista Lucio MdP. Metodología de la Investigación (6th ed.). Madrid, España: McGraw-Hill Interamericana de España; 2014.

[40] Casas L, Ahumada C. Teenage sexuality and rights in Chile: from denial to punishment. Reprod Health Matters. 2009;17(34):88–98. doi:10.1016/S0968-8080(09)34471-7 19962642

[41] Rojas Ramírez G, Eguiguren Bravo P, Matamala Vivaldi MI, et al. Adolescent access to contraception: perceptions of health workers in Huechuraba, Chile. Rev Panam Salud Públ. 2017;41:e77. https://iris.paho.org/handle/10665.2/34040. 10.26633/RPSP.2017.77 PMC6645252 28614485

[42] De Souza NMF, Rodrigues Selis LM. Gender violence and feminist resistance in Latin America. Int Femin J Polit. 2022;24(1):5–15. doi:10.1080/14616742.2021.2019483

[43] Revilla Blanco M. Del ¡Ni una más! al #NiUnaMenos: movimientos de mujeres y feminismos en América Latina. Política y Sociedad. 2019;56(1):47–67. doi:10.5209/poso.60792

[44] Bilbao BS. *El cuerpo como trinchera. Experiencias contrainformacionales y modos de configurar la resistencia desde la práctica política del feminismo argentino* 2019. Buenos Aires, Argentina: Universidad Nacional de Quilmes. http://unidaddepublicaciones.web.unq.edu.ar/libros/el-cuerpo-como-trinchera-experiencias-contrainformacionales-y-modos-de-configurar-la-resistencia-desde-la-practica-politica-del-feminismo-argentino/.

[45] Mills R, Northcott K, Kovacs E, et al. Opening a portal to pleasure based sexual and reproductive health around the globe: a qualitative analysis and best practice development study. Sex Reprod Health Matt. 2023;31(1):152–168. doi:10.1080/26410397.2023.2275838 PMC10833112 38037813

[46] Lindberg LD, Bell DL, Kantor LM, et al. The sexual and reproductive health of adolescents and young adults during the COVID-19 pandemic. Perspect Sex Reprod Health. 2020;52(2):75–79. doi:10.1363/psrh.12151 32537858 PMC7323157

[47] Ortiz Contreras J, Quiroz Carreño JM, Neira Contreras R, et al. Systematization of initiatives in sexual and reproductive health about good practices criteria in response to the COVID-19 pandemic in primary health care in Chile. Medwave. 2022;22(06):002555. doi:10.5867/medwave.2022.06.002555 35917236

[48] Khosla R, Mishra V, Singh S. Sexual and reproductive health and rights and bodily autonomy in a digital world. Sex Reprod Health Matt. 2023;31(4)):2269003. doi:10.1080/26410397.2023.2269003 PMC10629411 37930349

[49] Faúndes A, Távara L, Brache V, et al. Emergency contraception under attack in Latin America: response of the medical establishment and civil society. Reprod Health Matters. 2007;15(29):130–138. doi:10.1016/s0968-8080(07)29300-0 17512384

[50] Richardson E, Birn A-E. Sexual and reproductive health and rights in Latin America: an analysis of trends, commitments and achievements. Reprod Health Matters. 2011;19(38):183–196. doi:10.1016/S0968-8080(11)38597-7 22118152

[51] Craig SL, Eaton AD, McInroy LB, et al. Can social media participation enhance LGBTQ+ youth well-being? Development of the social media benefits scale. Soc Med Soc. 2021;7(1):1–13. doi:10.1177/2056305121988931

[52] Starrs AM, Ezeh AC, Barker G, et al. Accelerate progress—sexual and reproductive health and rights for all: report of the Guttmacher–Lancet Commission. Lancet. 2018;391(10140):2642–2692. doi:10.1016/S0140-6736(18)30293-9 29753597

[53] Bjorkman M, Malterud K. Lesbian women’s experiences with health care: A qualitative study. Scand J Prim Health Care. 2009;27(4):238–243. doi:10.3109/02813430903226548 19958064 PMC3413916

[54] Formby E. Lesbian and bisexual women’s human rights, sexual rights and sexual citizenship: negotiating sexual health in England. Cult Health Sex. 2011;13(10):1165–1179. doi:10.1080/13691058.2011.610902 21972785

[55] Andreani V, Ivankovic F, Díaz C. Prácticas sexuales no heteronormadas en mujeres: violencias y (des)atenciones ginecológicas. Revista Punto Género. 2023;19:01–35. doi:10.5354/2735-7473.2023.71208

[56] Mertehikian YA. Salud sexual-reproductiva y heteronormatividad: experiencias con los servicios de salud de jóvenes lesbianas y bisexuales en el Área Metropolitana de Buenos Aires [Presentation]. IX Jornadas de Sociología, Universidad de Buenos Aires; 2015.

[57] Obach A, Sadler M, Cabieses B, et al. Strengths and challenges of a school-based sexual and reproductive health program for adolescents in Chile. PLoS One. 2022;17(3):e0265309. doi:10.1371/journal.pone.0265309 35320306 PMC8942266

[58] Le Guen M, Schantz C, Régnier-Loilier A, et al. Reasons for rejecting hormonal contraception in Western countries: a systematic review. Soc Sci Med. 2021;284:114247. doi:10.1016/j.socscimed.2021.114247 34339927

[59] WHO. Global health sector strategies on, respectively, HIV, viral hepatitis and sexually transmitted infections for the period 2022-2030. World Health Organization; 2022.

[60] Smith J, Mallouris C, Lee K, et al. The role of civil society organizations in monitoring the global AIDS response. AIDS Behav. 2017;21:44–50. doi:10.1007/s10461-016-1579-3 27734168 PMC5706462

[61] Boydell V, Schaaf M, George A, et al. Building a transformative agenda for accountability in SRHR: lessons learned from SRHR and accountability literatures. Sex Reprod Health Matt. 2019;27(2):64–75. doi:10.1080/26410397.2019.1622357 PMC7942763 31533591

[62] Keen S, Lomeli-Rodriguez M, Joffe H. From challenge to opportunity: virtual qualitative research during COVID-19 and beyond. Int J Qual Methods. 2022;21: 1–11. doi:10.1177/16094069221105075 PMC9167989 35692956

